# Effect, process, and economic evaluation of a combined resistance exercise and diet intervention (ProMuscle in Practice) for community-dwelling older adults: design and methods of a randomised controlled trial

**DOI:** 10.1186/s12889-018-5788-8

**Published:** 2018-07-13

**Authors:** Ellen J. I. van Dongen, Annemien Haveman-Nies, Nick L. W. Wezenbeek, Berber G. Dorhout, Esmée L. Doets, Lisette C. P. G. M. de Groot

**Affiliations:** 1 Food, Health & Consumer Research, Wageningen Food & Biobased Research, P.O. Box 17, 6700 AA Wageningen, The Netherlands; 2 Division of Strategic Communication, Wageningen University and Research, P.O. Box 17, 6700 AA Wageningen, The Netherlands; 3 Division of Human Nutrition, Wageningen University and Research, P.O. Box 17, 6700 AA Wageningen, The Netherlands; 4 GGD Noord-en Oost-Gelderland, Academic Collaborative Centre AGORA, P.O. Box 3, 7200 AA Zutphen, The Netherlands

**Keywords:** Sarcopenia, Resistance exercise, Dietary protein intake, Community-dwelling older adults, Real-life setting, Evaluation, Physical functioning

## Abstract

**Background:**

Exercise and nutrition are important for older adults to maintain or to regain their muscle mass, function, strength, and ultimately quality of life. The effectiveness of combined resistance exercise and diet interventions is commonly evaluated in controlled clinical studies, but evidence from real-life settings is lacking. This article describes the effectiveness, process, and economic evaluation design of a combined nutrition and exercise intervention for community-dwelling older adults in a Dutch real-life setting.

**Methods:**

The ProMuscle in Practice study is a randomised controlled multicentre intervention study, conducted in five municipalities in the Netherlands. Two hundred community-dwelling older adults (≥65 years) who are frail or pre-frail based on Fried frailty criteria or who experience strength loss are randomised over an intervention and control group by municipality. In the first 12-week intensive support intervention, participants in the intervention group perform resistance exercise training guided by a physiotherapist twice a week and increase protein intake by consuming protein-rich products under the supervision of a dietitian. Afterwards, they continue with a 12-week moderate support intervention. The control group receives only regular care during the two 12-week periods. Effect outcomes are measured at all locations at baseline, 12 weeks, 24 weeks, 36 weeks and only at three locations at 52 weeks. The primary outcome is physical functioning (Short Physical Performance Battery). Secondary outcomes include leg muscle strength, lean body mass, activities of daily living, social participation, food intake, and quality of life. Qualitative and quantitative implementation process data are collected during the intervention. Healthcare use and intervention costs are registered for the economic evaluation.

**Discussion:**

Evaluating the effects, implementation, and costs of this combined intervention provides valuable insight into the feasibility of this intervention for community-dwelling older adults and into the intervention’s ability to improve or to maintain physical functioning and quality of life.

**Trial registration:**

Netherlands Trial Register (NTR6038) since 30 August 2016.

**Electronic supplementary material:**

The online version of this article (10.1186/s12889-018-5788-8) contains supplementary material, which is available to authorized users.

## Background

Age-related loss of muscle mass and function, also known as sarcopenia [1–3], is a major scientific and public health problem. Sarcopenia prevalence ranges from 1 to 29% for community-dwelling older adults [4]. This geriatric condition increases the risk of adverse outcomes, such as physical disability, lower quality of life, and mortality [1], and impacts the ability to live independently. Furthermore, sarcopenia greatly influences healthcare expenses: in the Netherlands healthcare costs of community-dwelling sarcopenic older adults are €11,000 higher per year than costs of non-sarcopenic older adults [5]. Metabolic changes, physical inactivity, and insufficient dietary intake are causal factors in the development of sarcopenia [1, 3].

There is accumulating evidence that sarcopenia can be counteracted with lifestyle changes. Reviews and meta-analyses have shown that interventions including resistance exercise (RE) and dietary strategies towards improving protein intake effectively increase muscle outcomes in older adults [6–9]. However, as these interventions are mostly implemented in highly controlled settings, no conclusions can be drawn about their effectiveness when implemented in a real-life setting. There are large differences between controlled clinical settings and real-life settings. In real-life settings, interventions are implemented by healthcare professionals working in a variety of organisations and settings, rather than by researchers. Therefore, some flexibility in implementation should be allowed in real-life settings [10] to account for the local context (i.e. organisation structure, responsibilities, capacity) and the needs of the target group. Slight deviations from the intervention protocol to tailor the intervention to the local setting are therefore likely. Consequently, there is a need to translate these efficacious clinical interventions to real-life healthcare and community settings and investigate their effectiveness in practice.

In a real-life setting therefore, a more extensive evaluation approach is required to show effectiveness when compared to a clinical efficacy study. The evaluation should focus on effect outcomes that are of interest for future implementers or stakeholders in order to increase the chances of implementation continuing after the effectiveness study. Furthermore, a process evaluation is needed to describe what happens during implementation, to explain intervention effects [11], and to allow continuous optimisation of implementation protocols. Lastly, healthcare costs related to intervention effects should be assessed in an economic evaluation, as this is important to support sustainable implementation of the intervention and to embed the intervention in the policy of care organisations or local governments. Although some studies on different physical activity and/or diet interventions in older adults include all three evaluation components [12, 13], most studies report only effect evaluations [14–16]. There is thus a lack of information on the other evaluation components for the implementation of a resistance exercise and diet intervention to counteract sarcopenia in practice. Therefore, we translated an effective resistance exercise and dietary protein nutrition intervention for community-dwelling older adults [17] to fit the practice setting [18]. As a next step, this paper describes the design of the multicentre effectiveness study on this adapted resistance-type exercise and nutrition intervention for community-dwelling older adults in Dutch healthcare practice. The objectives of this study are to examine 1) the effectiveness of a combined resistance exercise and nutrition intervention for community-dwelling older adults on i.e. physical functioning, muscle strength, muscle mass, quality of life, and social participation (effectiveness evaluation); 2) implementation integrity, acceptability, applicability, and dose received of the intervention (process evaluation); and 3) the cost-effectiveness of the ProMuscle in Practice intervention in a real life-setting, compared to usual care (economic evaluation).

## Methods/Design

### Study design

**Fig. 1:**
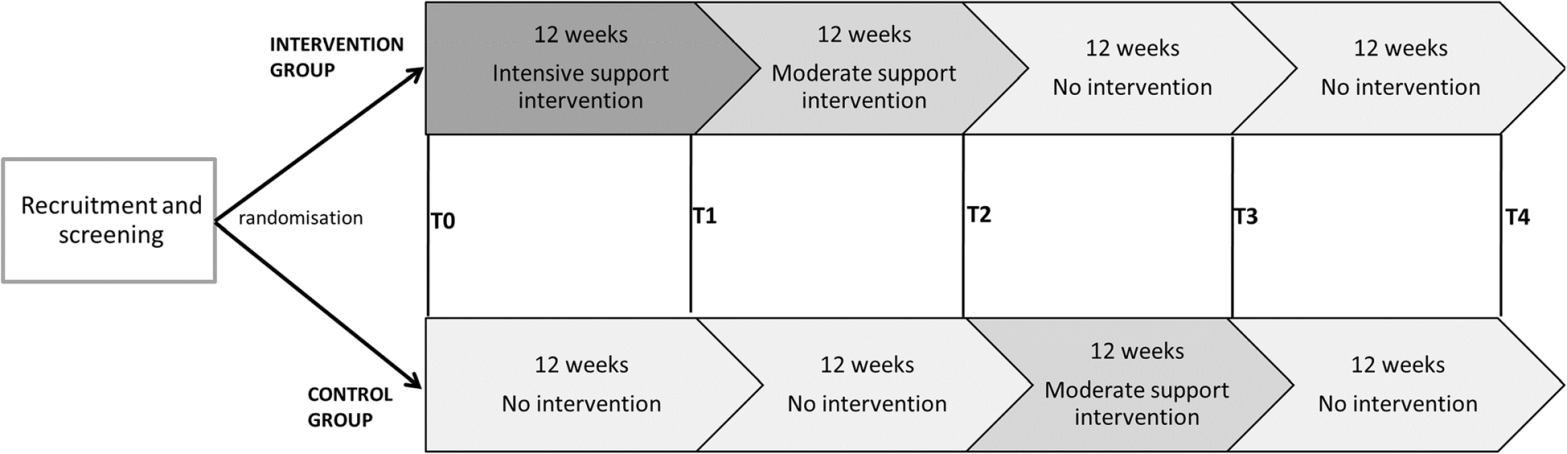
Study design and measurements (T0, T1, T2, T3, and T4) per intervention location. The 12-week intensive support intervention consists of resistance exercise training sessions twice a week under the supervision of a physiotherapist, focused on the major muscle groups, and increasing dietary protein intake to 25 grams per main meal under the supervision of a dietitian. The moderate support intervention comprises optional resistance exercise sessions at local facilities (e.g. fitness centre or sports hall) and five group-based nutrition workshops. T0, T1, T2, and T3 measurements are taken in all five intervention municipalities, T4 measurements are performed only in Epe, Ermelo/Putten, and Harderwijk

This study is a randomised controlled multicentre intervention study, in five different municipalities in the Netherlands. The duration of the study is 36 weeks in two municipalities (Apeldoorn and Ede) and 52 weeks in three municipalities (Epe, Ermelo/Putten, and Harderwijk). The intervention comprises resistance exercise training with a focus on the leg muscles and a diet intervention focused on increasing protein intake. For the intervention group, this includes a 12-week intensive support intervention period (weeks 1–12) followed by a 12-week moderate support intervention period (weeks 13–24). The control group receives no intervention (weeks 1–24) to allow comparison with the intervention group in this period, followed by the delayed moderate support intervention (weeks 25–36). Participants receive no additional support after the 24-week intervention period in the intervention group and the 12-week intervention period in the control group, see Fig. 1. Effect measures and healthcare cost measures are performed every 12 weeks, and process measures are performed continuously during the study. The ProMuscle in Practice study has been registered at Netherlands Trial Register (NTR6038) since 30 August 2016. The Wageningen University Medical Ethics Committee approved the study protocol and all participants provide written informed consent before the start of the study.

### Setting

The study is carried out in five municipalities in the province of Gelderland, the Netherlands. These include three small cities (10,000–100,000 inhabitants: Epe, Ermelo/Putten, and Harderwijk) and two cities (> 100,000 inhabitants: Apeldoorn and Ede). The intensive support intervention is delivered by healthcare professionals from four regional care organisations (Zorggroep Apeldoorn, Viattence, Zorggroep Noordwest-Veluwe, and Opella). The moderate support intervention is designed by the community health service in collaboration with the selected municipalities and local organisations, such as a sports-promoting agency or prevention centre. These local organisations and the municipal health service deliver this moderate support intervention.

### Sample size calculation

The sample size calculation is based on the difference in change in Short Physical Performance Battery (SPPB) score between the intervention and control group after 12 weeks in the experimental ProMuscle trial of 1.2 with a standard deviation of 1.4 [17]. Because the current study is performed in a real-life setting instead of a highly controlled research setting, only 75% of the previously observed change in SPPB score is expected. Furthermore, we take into account a drop-out of 30% within the first 12 weeks. Assuming an alpha of 0.05, power of 90%, and a two-sided test, a sample size of 78 participants per group is required. To account for clustering effects, we aim for 100 participants per research group. Participants are equally divided over the five locations, so each location should provide 40 participants (i.e. 20 intervention and 20 control participants).

### Study population and recruitment

The study population consists of community-dwelling older adults, 65 years or over, from the selected municipalities (Apeldoorn, Epe, Ermelo/Putten, Harderwijk, and Ede). Participants are mainly recruited through announcements and adverts in local newspapers, posters in public spaces and meeting centres, via homecare nurses of the care organisations, and in collaboration with local organisations for older adults. Recruitment strategies may differ between the different intervention locations. All interested older adults receive an extensive information brochure and are invited to an information meeting. If they remain interested, they are invited for a screening visit in their municipality to evaluate eligibility for study participation based on the inclusion and the exclusion criteria (Table 1). After signing an informed consent, potential participants complete Fried’s frailty test [19], a medical questionnaire, and the 4-item Simplified Nutritional Appetite Questionnaire (SNAQ) [20]. If a person is non-frail, an additional screening questionnaire is administered to check whether this person experiences difficulty in daily activities due to loss of muscle strength. If a person fits the inclusion criteria, that person’s general practitioner (GP) performs a check on eligibility based on the exclusion criteria. The GP informs the researchers whether the person can participate safely, and, if the GP approves, the researchers include the person in the study. After inclusion, participants are randomly allocated to the intervention or the control group at each location, stratified by gender and frailty status. Couples are allocated to the same group to prevent contamination. The researchers randomise the participants based on a randomisation scheme constructed by an independent person from the division of Human Nutrition of Wageningen University (Netherlands).

**Table 1:** Inclusion and exclusion criteria for the ProMuscle in Practice study

Inclusion criteria	Aged 65 years or over
	Living independently in one of the selected municipalities (Apeldoorn, Epe, Ermelo/Putten, Harderwijk, Ede)
	Mastery of the Dutch language
	Meet one of the two following criteria: - Score 1 or more points on the Fried frailty criteria [19] - Do not perform whole body resistance exercises for > 30 min on 2 or more days per week, and report loss of muscle strength
	Having signed informed consent
Exclusion criteria	Having an allergy to, or being sensitive to, milk proteins or being lactose intolerant
	Diagnosed COPD or cancer
	Diagnosed diabetes type 1 or type 2, that is unstable, not well regulated with medication, or the participant is not able to notice hypoglycaemia
	Diagnosed hypertension (systolic blood pressure > 160 mmHG) that is not well regulated with medication
	Severe heart failure
	Renal insufficiency (eGFR < 30 ml/min)
	Having physical impairments that prevent them from participating in the exercise training
	Having cognitive impairments that prevent them from understanding and completing questionnaires
	Receiving terminal care
	Having a newly fitted artificial hip or knee prosthesis, unless fully recovered
	Having recent surgery (< 3 months) scars that the exercises might stress

### Logic model

**Fig. 2:**
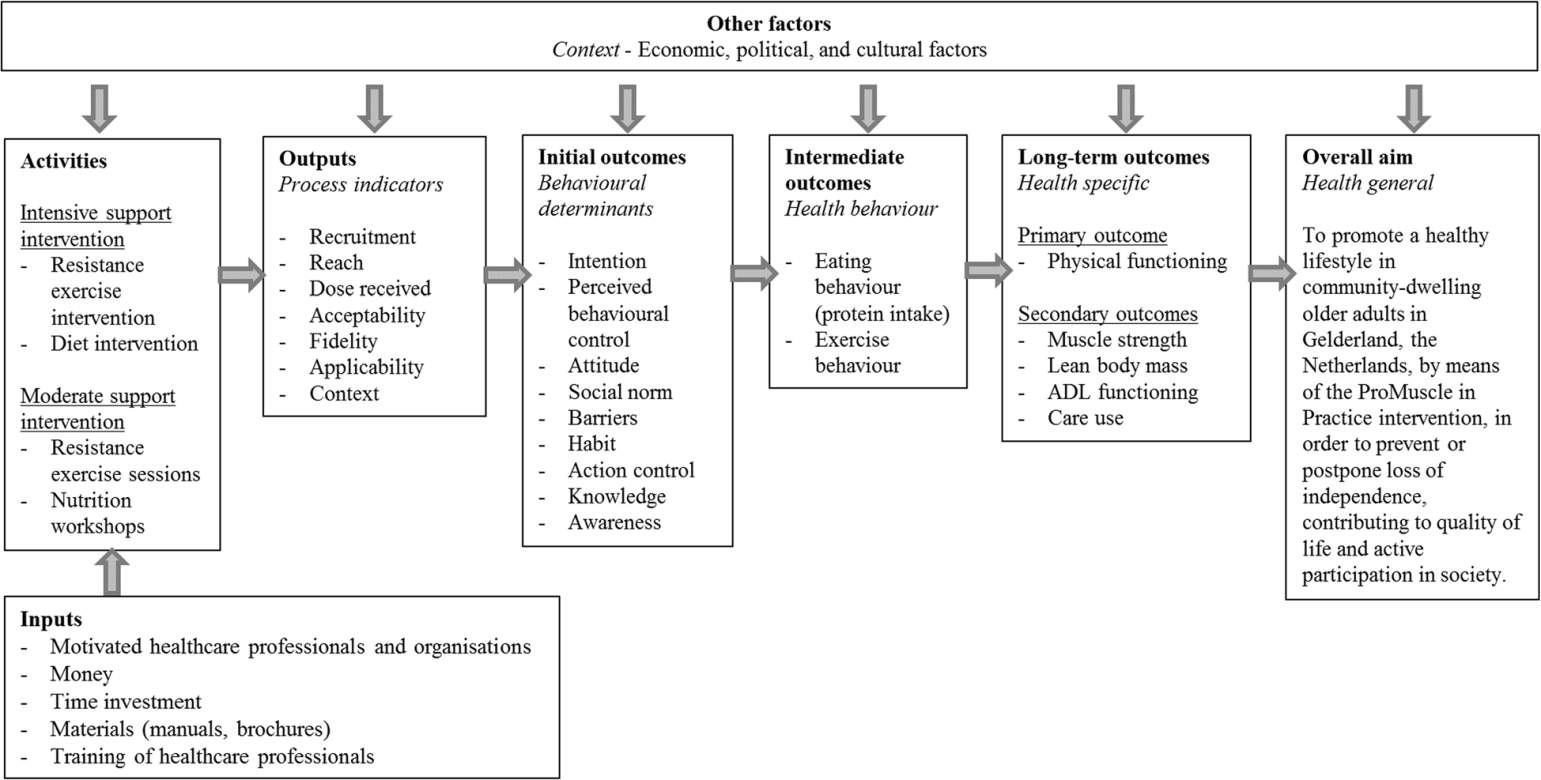
Logic model of change for the ProMuscle in Practice intervention

We created a logic model for the intervention (Fig. 2) showing intervention activities and their proposed mechanism of change in outcomes such as behaviour or health [11]. Adequate implementation of the intervention activities is expected to improve dietary and exercise behaviour (intermediate outcomes), which in turn will affect health-specific long-term outcomes such as physical functioning and muscle strength. The overall aim of the intervention is to prevent or postpone loss of independence and to contribute to quality of life.

### Intervention

The intervention consists of an intensive support intervention (12 weeks, intervention group only) and a moderate support intervention (12 weeks, separately for the intervention group and the control group).

#### Intensive support intervention

The intensive support intervention is based on an efficacious clinical trial [17] and adapted to fit the real-life setting [18]. The adapted intervention uses a combination of behaviour change methods for the healthcare professionals (HCP) and participants, such as tailoring, persuasive communication, and self-monitoring [18], see also Additional file 1. The intensive support intervention is implemented by physiotherapists and dietitians from local care organisations. The researchers provide a 1 h general information meeting, a more detailed 1.5 h training session, and detailed implementation manuals to the HCPs before the intervention starts. If desired, HCPs can contact HCPs in another intervention location for additional information, e.g. tips and tricks for implementing the resistance exercise training. Halfway through the programme, HCPs at each location have a joint peer discussion. Furthermore, the research team functions as a helpdesk during the trial. For an extensive overview of core HCP tasks and behaviour change methods, see Additional file 1.

##### Resistance exercise intervention

Under the supervision of physiotherapists, participants undertake progressive resistance exercise training twice a week. Each training session lasts 1 hour, and training groups consist of about six participants. Every training session starts with a warm-up using a home trainer (bike) for 5 minutes or a warm-up under the physiotherapist’s guidance. Afterwards, participants perform exercises using the following machines: leg press, leg extension, lat pulldown, vertical row, and chest press (Technogym BV, Rotterdam, The Netherlands) to target the major muscle groups. The session ends with a group-based warm-down including stretch exercises. The objective of the resistance exercise intervention is that participants increase the training load for the leg exercises from 50% of their one-repetition maximum (1-RM) (four sets of 10–15 repetitions) at baseline to 75% of their 1-RM (four sets of 8–12 repetitions) in weeks 7 to 12. Before the intervention starts, participants perform a maximum strength test on the leg press and the leg extension machine. The physiotherapists use the outcome of this test to tailor individual resistance-exercise programmes. Participants perform the other exercises at a lower intensity up to a maximum of approximately 60% of their 1-RM (three sets of 15 repetitions), with optional small increases in training load. Physiotherapists can add exercises to train coordination or balance, but most emphasis should be placed on progressively training the leg muscles. The physiotherapists should also ensure that participants take enough rest between exercises and check regularly whether participants have any problems or complaints. If a participant has complaints or injuries, physiotherapists are allowed to deviate from the training protocol provided. At week 6, physiotherapists test the maximum leg strength (3-RM) again and recalculate this to the 1-RM. Physiotherapists can use this 3-RM to monitor progression in training and to evaluate the training programme with the participants.

##### Diet intervention

The objective of the dietary intervention is to ensure that participants have a protein intake of at least 25 g at each main meal (breakfast, lunch, and dinner). Before the start of the 12-week intervention, the dietitian formulates tailored advice based on a 3-day food diary. Dietitians provide this tailored advice during an individual 30-min intake consultation, while also discussing regular dietary habits and preferences. The dietitian recommends mainly dairy-based protein-rich products, such as cheese, drinks, yoghurt. These are provided for free during these 12 weeks. These products can be used in addition to the regular diet, or as a substitute for dietary components, and are meant to help the participants increase daily protein intake. Participants receive these products each week during one of the training sessions. The first time the products are handed out, the dietitian is present to provide additional explanations or to answer questions. Around week 6 of the intervention period, the dietitian has an individual 15-min evaluation consultation with the participants to discuss experiences, possible complaints, and how participants can maintain the increased protein intake after the end of the first 12-week period. The dietary advice can be adjusted if needed, and the participants’ weight is also monitored. During the 12-week intervention period, participants are asked to indicate on a checklist whether they have consumed the recommended protein-rich products. They hand in this checklist every week at the training session, and the dietitian can use these checklists to monitor compliance with consuming the recommended products and see whether an additional (phone) consultation is needed.

#### Moderate support intervention

The intervention group starts the moderate support intervention after the intensive intervention. The control group receives this moderate support intervention only after 24 weeks of being a regular care control group, without receiving the intensive support intervention. The aim of the moderate support intervention is to encourage participants to continue consuming sufficient protein at main meals and engaging in resistance exercise training. About 4 weeks before the moderate support intervention starts, participants receive an information leaflet that includes information on available activities including both exercise sessions and dietary workshops, and suggestions about including (home) exercises and protein-rich products in their daily routine. Healthcare professionals from the intensive support intervention encourage the intervention group to participate. Participants could choose to join all, some, or none of the activities offered.

##### Resistance exercise sessions

Group exercise sessions take place twice a week at local sports clubs, gyms, or in collaboration with care sport connectors (brokers whose role is to connect the primary care and the sports sector). The trainers offer an exercise programme that includes strength exercises focusing on the legs, based on a manual designed for the moderate support intervention. The exercise sessions are group based and under professional guidance. Financial support for the moderate support intervention may be provided by municipalities or organisations, and participants have to pay nothing or a reduced price for the exercise sessions. At one or more meetings before the sessions start, the municipal health service instructs the trainers who implement the exercise sessions. Trainers also receive an implementation manual for this moderate support intervention. The trainers and the municipal health service have a midterm evaluation meeting before the control group starts the intervention. The municipal health service and the research team also serve as a helpdesk during the intervention period.

##### Nutrition workshops

Five 1.5-h nutrition workshops are organised in each municipality by the municipal health service, based on a newly developed course guide. During these workshops, participants receive information on how to incorporate protein-rich foods in their diet, share experiences, cook and taste protein-rich meals (breakfast, lunch, dinner), visit a supermarket (optional), and can experiment with a newly developed e-health app. The nutrition course is offered free of charge for both study groups. Intervention participants no longer receive free protein-rich food products. These workshops are implemented by a health promotion employee of the municipal health service, in collaboration with a dietitian to answer nutrition-related questions. As the health promotion employee is involved in designing the workshops, no additional training is provided for this intervention.

##### Newsletter

Once participants receive the intensive or moderate support intervention, they also receive a bi-monthly newsletter via e-mail, sent out by the municipal health service. The newsletter includes information about the study and interventions at the different locations, and stories from study participants or researchers.

### Outcomes

All participants are measured at baseline (T0), after 12 weeks (T1), after 24 weeks (T2), and after 36 weeks (T3). A selection of outcomes is also measured after 52 weeks (T4) at three intervention locations. At T0, T1, and T2, participants visit the research location in Wageningen once in the morning and the research location in their municipality once in the afternoon (on different days). The T3 and T4 measures are taken during one afternoon visit in their municipality. Participants are invited for the measurements by regular mail and are phoned if necessary. Participants receive a small financial compensation after completion of the final measurement. Un-blinded trained researchers and assistants take the measurements according to standardised protocols. Table 2 provides an overview of outcomes, indicators, methods, and time points.

**Table 2:** Overview of indicators, methods, and time points of data collection

			-T1 (Enrolment)	T0 (week 0)		T1 (week 12)		T2 (week 2)4		T3 (week 3)6		T4^c^ (week 52)	
Enrolment	Indicators	Method		INT^a^	CON^b^	INT	CON	INT	CON	INT	CON	INT	CON
		Informed consent	X										
Eligibility screen	Frailty state^d^	Fried frailty criteria [19], medical questionnaire, additional screening questionnaire (optional)	X										
Allocation				X	X								
Outcomes	Indicators	Method											
Socio-demographics	Age, gender, education, ethnic background, marital status, job status, smoking	Participant questionnaire [21]		X	X								
	Disease history	Participant questionnaire [21]		X	X								
	Height	Stadiometer		X	X								
	Nutritional status^d^	SNAQ [20]	X										
	Olfactory function	Sniffin’ sticks [22]		X	X								
	Meal functionalities	Questionnaire [23]		X	X								
Overall	Quality of life	EQ-5D-5L [25]^e^		X	X	X	X	X	X	X	X	X	X
Long-term	Physical functioning / fitness	SPPB [27], TUG [28, 29], 6MWT [30]		X	X	X	X	X	X	X	X	X	X
		Basic Lower Extremity function questionnaire [32]		X	X	X	X	X	X		X		
	Lower extremity strength	3-RM on leg press and leg extension		X	X	X	X						
		Knee extension with hand held dynamometer (MicroFET)		X	X	X	X	X	X	X	X	X	X
	Body composition (lean mass, fat mass, hydration status) and weight	DXA, BIS		X	X	X	X	X	X				
		Weighing scale		X	X	X	X	X	X	X	X	X	X
	Social participation	Social Role Domain questionnaire [36]		X	X	X	X	X	X		X		
Intermediate	Dietary / protein intake	3-day food diaries		X	X	X	X	X	X		X		
		Urinary nitrogen^f^		X	X	X	X	X	X				
	Physical activity	LAPAQ [37]		X	X	X	X	X	X		X		
		Actigraph^g^		X	X								
Initial	Behavioural determinants	Participant questionnaire (based on [38–45])		X	X	X	X	X	X				

^a^Intervention participants

^b^Control participants

^c^Only in Epe, Ermelo/Putten, and Harderwijk

^d^Measured during screening

^e^Also collected through regular post at T0.5 (week 6) and T1.5 (week 18)

^f^Collected once at one of the time points for each participant, not collected in Apeldoorn

^g^Collected in a random subsample of participants

#### Socio-demographic characteristics

Socio-demographic characteristics are assessed at baseline through a questionnaire based on The Development of the Older Persons and Informal Caregivers Survey Minimal DataSet (TOPICS-MDS) questionnaire [21], including questions on age, gender, education level, ethnicity, living situation, marital status, dental or swallowing problems, receiving formal or informal care, diseases, smoking, alcohol consumption, history of physical activity, and (past) occupation. Participant height is measured at baseline only, participant weight is collected at all time points. Weight and height are measured twice, and if there is too much disagreement between the two measures (> 0.1 kg or > 0.3 cm), a third measure is performed. Body Mass Index (BMI) is calculated from these measures. Olfactory function is checked at baseline using the Sniffin’ Sticks odour identification test [22]. At baseline, participants are asked to indicate the main functionalities associated with meals consumed at breakfast or lunch on a 26-item questionnaire based on questions from den Uijl et al. [23]. Protein intake is validated by urinary nitrogen from a single 24-h urine sample. All participants without incontinence problems from four intervention locations are asked to collect their urine once, on one of the days they fill in the food diary (either at T0, T1, or T2). Urine completeness is checked using the Para-AminoBenzoic Acid (PABA) marker [24].

#### Effectiveness evaluation

##### Overall outcome

Quality of life is measured by the EQ-5D-5L questionnaire [25], completed at T0, T0.5 (week 6), T1, T1.5 (week 18), T2, T3, and T4. This questionnaire is used to calculate Quality Adjusted Life Year (QALY) [26]. Additionally, a Visual Analogue Scale (VAS) is used to assess perceived health (scale 0–100), with 100 being the best possible health.

##### Long-term outcomes

The primary outcome of this study is the Short Physical Performance Battery (SPPB), a measure of physical functioning including three aspects: standing balance, gait speed, and a repeated chair rise test [27]. Two other tests of physical functioning are included; the Timed Up-and-Go test (TUG, [28, 29]) and the 6 minute walking test (6MWT) [30]. The 6MWT is a measure of fitness, and the number of metres walked in 6 minutes on a straight track of 10 m is recorded. The use of a walking aid is permitted in all three tests and should then be used at all time points. The SPPB, TUG, and 6MWT are measured at T0, T1, T2, T3, and T4 in both groups.

Lower extremity muscle strength is measured through 3 Repetition Maximum tests (3-RM) at T0 and T1, on both a leg press and a leg extension machine (Technogym BV, Rotterdam, The Netherlands). At baseline, first a familiarisation session including a maximum strength estimation test is performed, and a week later a maximum strength confirmation test is performed, aiming to achieve a 3-RM. The 3-RM confirmation scores (kg) are recalculated to 1 Repetition Maximum (1-RM), based on Brzycki’s formula [31]. Additionally, at T0, T1, T2, T3, and T4, knee extension force is measured using a hand-held dynamometer (MicroFET) with belt-stabilisation of the lower leg. A male researcher performs three repeated tests alternating both legs to define maximum strength in Newton.

Body composition is measured through total-body Dual Energy X-ray Absorptiometry (DXA) scans (Lunar Prodigy Advance, GE Health Care, Madison, WI). Total body lean mass, appendicular lean mass (sum of leg and arm lean mass), and fat mass are used as outcomes. Additionally, hydration status is assessed by Bio Impedance Spectroscopy (BIS, using a SFB7 impedance analyser from ImpediMed Limited, Pinkenba QLD, Australia). The BIS and DXA are conducted in the morning at T0, T1, and T2. Participants are asked to consume a standardised, light breakfast on the scan days and to defecate just before the measurements.

Activities of daily living (ADL) and social participation are measured at T0, T1, and T2 in both groups, and at T3 in the control group. ADL is measured through the Late Life Functional Disability Index related to Basic Lower Extremity Function [32]. Fourteen daily activities can be scored on a 5-point scale, ranging from ‘no difficulty’ to ‘I cannot do this’. Three additional items are included for participants who use a walking aid (e.g. a walker). The scores obtained for each question are added to a total raw score that equals a scaled score of basic lower extremity function; the higher the score, the better the ADL function [33]. Furthermore, participants complete the 5-item SARC-F questionnaire [34] and an additional question on knee pain from the Knee injury and Osteoarthritis Outcome Score questionnaire [35].

Social participation is measured through the Social Role Domain questions of the Late Life Functional Disability Index [36]. The questionnaire includes 16 items that ask both the frequency of performing different social activities (5-point scale ranging from ‘very often’ to ‘never’) and the difficulty participants perceive performing those activities (5-point scale ranging from ‘not at all’ to ‘very much’). Similar to the ADL questions, a total score is calculated that equals a scaled score [33]. The ADL questionnaire and the Social participation questionnaire have been translated to Dutch and were pretested in an older adult population (*n* = 5 and *n* = 6, respectively).

##### Intermediate outcomes

Dietary intake, with a special interest in protein intake, is measured through 3-day food diaries. Participants receive written and verbal (telephone) instructions and complete the diaries on three randomly allocated days (two weekdays [Monday–Thursday] and one weekend day [Friday–Sunday]). At T0, a trained research dietitian visits the participants at home, preferably within a week of completing the diary. The diary is checked, and measures are taken from common household items that people use to consume protein-rich foods (e.g. glasses, cups), according to a standardised protocol. At all other time points, diaries are checked by telephone by a trained research dietitian within a week of completion. Food consumption data are coded (type of food and amount) and energy and macronutrient intakes are calculated with Compleat (food calculation programme developed by the Division of Human Nutrition, Wageningen University). Additionally, a question on (vitamin D) supplement use is included.

Physical activity is measured by the LASA Physical Activity Questionnaire (LAPAQ) [37]. Additionally, Accelerometers (Actigraph GT3X) are used in a random subsample of participants at baseline, who were asked to wear the accelerometers on their hip for seven consecutive days.

##### Initial outcomes

Participants complete a self-developed questionnaire on behavioural determinants of dietary protein intake at T0, T1, and T2. Behaviour is formulated as ‘eating protein-rich products at breakfast and lunch’. Items to measure intention, perceived behavioural control, attitude, and social norms are based on scales described in the literature [38–41]. Items on barriers to eating protein-rich foods are based on items formulated to assess barriers related to physical activity [42]. Items to assess habits are adapted from the Self Report Index of habit strength [43]. Action control items are based on questions used by den Braver et al. [44]. To assess knowledge, participants are asked to indicate whether products frequently consumed by older adults (informed by Ocké et al. [45]) are rich in protein. Additionally, awareness of protein-rich foods and health is assessed with two items. This questionnaire has been pre-tested in a sample of older adults (*n* = 4).

#### Process evaluation

Data from both participants and healthcare professionals are collected to assess intervention implementation at the different locations for both the intensive support intervention and the moderate support intervention. This evaluation is guided by the RE-AIM framework, the Medical Research Council guidelines for process evaluation [11], and the Conceptual model for implementation research [46]. Process measures include the indicators recruitment, reach, dose received, acceptability (for implementers and participants), fidelity, applicability (appropriateness or feasibility), and context [11, 46–51]. Process evaluation methods include a project logbook, registration forms, and attendance lists completed by HCPs, participant questionnaires (T0, T1, T2, T3, and T4) and semi-structured interviews (T2 and T3), semi-structured interviews with HCPs (T1 and T3), and structured observations of the intervention components (between T0 and T3).

#### Economic evaluation

For the economic evaluation, additional information on care use and costs is collected. Participants complete a questionnaire to assess healthcare utilisation, participant out-of-pocket costs, and productivity losses, the latter based on the Productivity Cost Questionnaire [52]. To facilitate recall during measurements, participants record their care use in a cost diary in the period between measurements. The direct and indirect healthcare costs are recalculated using the standard prices for cost research in healthcare, provided by the Dutch Healthcare Institute [53]. Outcomes of the SPPB and the EQ-5D (QALY) are used to assess incremental cost-effectiveness and cost-utility, respectively. Intervention costs are registered by the researchers and the involved HCPs from the care organisations (type and duration of care provided).

### Statistical analysis

Quantitative data analyses are performed using the intention-to-treat principle. Descriptives are presented as mean and standard deviation, mean and 95% confidence interval, or percentage. If necessary, not normally distributed data are transformed. Linear mixed model analysis is used to assess differences in changes between the intervention group and the control group, with a significance level of 0.05. Analysis is adjusted for possible differences between the two groups at baseline and other possible confounders. Additionally, subgroup analysis is performed (e.g. per-protocol analysis or based on frailty state or socio-economic background).

Qualitative data (interviews) are audiotaped and transcribed verbatim. Transcripts are checked before analysis and are analysed using an inductive approach in ATLAS.ti.

For the economic evaluation, an incremental cost-effectiveness ratio (ICER) is calculated using a bootstrap analysis, based on costs and effects, in an analysis with a societal and healthcare perspective. In the societal perspective, all costs and benefits of the intervention are included, irrespective of who pays and who gets the benefit [54]. Cost-effectiveness planes and cost-effectiveness acceptability curves are plotted. Additionally, sensitivity analysis is performed.

## Discussion

This article described the comprehensive approach to evaluate a combined exercise and nutrition intervention to prevent sarcopenia in a real-life setting, including an effectiveness, a process, and an economic evaluation. The intervention focuses on resistance exercise for the major muscle groups and the consumption of at least 25 g of protein at the three main meals. It comprises an intensive support intervention and a moderate support intervention. The intensive support intervention is aimed at initiating behaviour change under the supervision of healthcare professionals, whereas the moderate support intervention provides support to sustain the behaviour change, making use of local facilities. To our knowledge, this is the first multi-component evaluation of a combined dietary and exercise intervention for community-dwelling older adults in a real-life setting.

When an intervention is being tested in a real-life setting, the ultimate aim is to enable its broad dissemination once effectiveness is shown. To achieve that, besides being effective and cost-effective, an intervention must be shown to be acceptable and feasible in order to achieve citizen and stakeholder support and structural financing. We have, therefore, included a broad range of effectiveness outcomes that are relevant for research, policy, and practice. The process evaluation adopts a mixed-methods approach, combining information from questionnaires, interviews, registration forms, and observations. This extensive process evaluation approach is expected to provide a clear insight into the delivery of the intervention and why this intervention is or is not effective in improving outcomes. With this, the intervention can be further improved to facilitate future implementation and dissemination. Furthermore, this study design allows us to make multiple comparisons of effects within one study. As most interest lies in the effectiveness of the combination of the intensive support intervention and the moderate support intervention during the first 24 weeks, we only include a control group in that period. The control group receives the moderate support intervention after these 24 weeks. By offering this, we allow the control group also to benefit from the intervention, and it also allows us to gain valuable information regarding the effectiveness of this less intensive intervention on the study outcomes. Furthermore, the follow-up measurements 12 or 24 weeks after the end of the moderate support intervention provide insight into the intervention’s long-term effects.

We aim to investigate effects and costs in physically frail older adults, as we expect frail older adults to benefit most from this intervention. We know that reaching and recruiting frail older adults for studies is challenging [55], and our pilot study showed that the intervention seems beneficial for a less frail population also [18]. Therefore, we include a broader population of older adults who are not necessarily frail but who do experience loss of muscle strength. Furthermore, the study population will probably include individuals who are highly motivated to change their dietary and exercise behaviour. This might be beneficial for compliance but also induces selection bias, making it more difficult to generalise findings to the overall population of Dutch community-dwelling older adults. As we expect differences between the different municipalities, we randomise participants per intervention location. In this way, we aim to achieve an overall comparable intervention and control group. A downside of this design is that participants randomised into the control group might potentially refrain from participation or change their exercise or dietary habits by themselves, even though we ask them not to do so.

The moderate support intervention is a newly developed programme that has not yet been tested on acceptability, feasibility, or effectiveness. The content of this programme is more practice-based, and no detailed implementation guidelines are used. We expect more variation in the implementation and content of the moderate support intervention between the different locations than in the implementation and content of the intensive support intervention. This makes it challenging to compare the effectiveness of the overall moderate support intervention. Therefore, this moderate support intervention receives extra attention in the process evaluation. This allows us to describe in detail how the intervention is tailored to the different contexts [11] and to obtain insight into best practices and factors for success or failure.

In conclusion, this study will provide valuable insight into the effectiveness, implementation, and cost-effectiveness of a combined exercise and nutrition intervention for community-dwelling older adults in five real-life settings. The results of this study are relevant for large-scale dissemination and implementation of this intervention in practice.

## Additional files

Additional file 1: Overview of the ProMuscle in Practice intensive support intervention and the moderate support intervention. (DOCX 43 kb)

## Abbreviations

1-RM: One-repetition maximum
3-RM: Three-repetition maximum
6MWT: Six minute walking test
ADL: Activities of daily living
BIS: Bio Impedance Spectroscopy
BMI: Body Mass Index
DXA: Dual Energy X-ray Absorptiometry
GP: General practitioner
HCPs: Healthcare professionals
ICER: Incremental cost-effectiveness ratio
LAPAQ: LASA Physical Activity Questionnaire
PABA: Para-AminoBenzoic Acid
QALY: Quality Adjusted Life Year
RE: Resistance exercise
SNAQ: Simplified Nutritional Appetite Questionnaire
SPPB: Short Physical Performance Battery
TOPICS-MDS: The Development of the Older Persons and Informal Caregivers Survey Minimal DataSet
TUG: Timed Up-and-Go test
VAS: Visual Analogue Scale
