# Supplementary material for: Effect, process, and economic evaluation of a combined resistance exercise and diet intervention (ProMuscle in Practice) for community-dwelling older adults: design and methods of a randomised controlled trial
Source: BMC Public Health. 2018 Jul 13;18:877. doi: 10.1186/s12889-018-5788-8 (PMC6045872; doi:10.1186/s12889-018-5788-8)
Supplement: Supplementary file 1 — Overview of the ProMuscle in Practice intensive support intervention and the moderate support intervention. (DOCX 43 kb) [file 12889_2018_5788_MOESM1_ESM.docx]

**Additional file 1. Overview of the intensive support intervention and the moderate support intervention**

**Intensive support intervention**

**DIETITIAN**

**Objective of the diet intervention:**

To advise and guide participants about consuming extra dietary protein in their daily dietary pattern, achieving intakes of 25–30 grams of protein with each main meal.

**Key attention points for the dietitian:**

- Provide insight into options to increase dietary protein intake;
- Remove barriers for participants regarding adequate dietary protein consumption;
- Empower and strengthen participants’ self-efficacy;
- Motivate participants to consume extra dietary protein.

**Core tasks of the dietitian:**

1. Perform intake consultation (30 minutes) and provide advice to participant

- 1. Broadly discuss the dietary intake pattern based on the completed 3-day food diary;
  2. Discuss the participant’s complaints regarding nutrition and digestion;
  3. Explain relation between dietary protein intake and physical functioning;
  4. Measure participant’s body weight;
  5. Provide advice concerning dietary protein intake, taking the participant’s preferences into account, and explain when to consume which protein-rich products;
  6. Explain how to fill in calendar to monitor compliance;
  7. Explain that the project has a duration of 24 weeks and that the participant should aim for long-term behaviour change.

2. Additional contact moment the first time that participants receive protein-rich products

1. Hand out the bag with the advised protein-rich products;
2. If needed, answer participant’s questions and repeat the tailored advice, including information on portion sizes and when to consume which protein-rich product.

3. Signal problems and non-compliance

1. Signal problems and non-compliance by checking the calendars every two weeks;
2. If needed, discuss problems and experiences in a (phone) consultation;
3. Motivate participant to consume protein-rich products and prevent drop-out;
4. Provide clear information and instructions on, and assess facilitators and barriers for, consuming protein-rich products.

4. Perform midterm evaluation consultation (15 minutes) and prepare participants for the moderate support intervention period

1. Evaluate the previous six weeks with the participant concerning consumption of protein-rich products;
2. Discuss experiences and compliance with the dietary advice with the participant, including an explanation that it is not desirable to compensate for the protein-rich products at mealtimes;
3. If relevant, discuss complaints concerning the consumption of the protein-rich products;
4. Measure participant’s body weight and check potential weight change;
5. If needed, adjust the advice concerning dietary protein intake;
6. Motivate participant to consume protein-rich products and prevent drop-out;
7. Explain the nutrition workshops within the moderate support intervention period in more detail and discuss with the participant how to independently maintain a protein-rich dietary intake pattern.

**PHYSIOTHERAPIST
Objective of the resistance exercise intervention:**

To supervise participants during the performance of progressive resistance-type exercises, working from 50% of 1 Repetition Maximum (1-RM) to 75–80% of 1-RM. The physiotherapist tailors the training sessions to the participants’ physical possibilities and motivates them to perform the exercises correctly.

**Key attention points for the physiotherapist:**

- Train participants’ load ability by increasing the training load. Attention should be paid to the balance between load ability and training load;
- Supervise resistance-type exercise sessions, with exercises focused on the major muscle groups;
- Remove barriers for participants;
- Motivate participants and promote having fun during exercise;
- Improve group coherence;
- Empower and strengthen participant’s self-efficacy;
- Provide ideas about being more physically active in daily life;
- Inform participants about the moderate support intervention on time and motivate them to participate.

**Core tasks of the physiotherapist:**

1. Map physical possibilities, complaints, or constraints of participants

1. Make an inventory of the participants’ expectations concerning the training sessions;
2. Analyse motivation and potential constraints for participating in the training sessions;
3. Provide a good introduction and familiarise participants with the machines, ensure that they feel comfortable doing the exercises.

2. Design and implement the ProMuscle in Practice resistance-exercise intervention

1. Ensure that there are at least two days of rest between the two training sessions;
2. Take individual participants’ load ability into account when starting the intervention;
3. Take the training protocol as guideline when performing the progressive exercise intervention;
4. Stimulate and motivate participant, provide positive feedback;
5. Answer participant’s questions about the intervention or discuss these questions with the dietitian;
6. Motivate participant to be physically active in daily life;
7. Stimulate group feeling, e.g. by performing a group-based warm-down;
8. Collect the calendars every week and pass them on to the dietitian.

3. Perform an intermediate evaluation (week 6)

1. Perform a maximum strength test (3-RM) with the participant and tailor the training protocol accordingly. Inform participants of their progress;
2. Discuss participant’s experiences with the training sessions during the intervention period;
3. Motivate participant to perform the resistance exercises and prevent drop-out;
4. Explain the content of the moderate support intervention to the participants and motivate them to continue with resistance exercises sessions or give tips on being physically active.

**Moderate support intervention**

**NUTRITION WORKSHOPS**

**Objective of the nutrition workshops:**The nutrition workshop leader will discuss theory about nutrition, facilitate the exchange of experiences with nutrition between participants, facilitate creating and tasting different protein-rich meals, and discuss homework assignments.

**Course content of the individual nutrition workshops:**

| **Workshop** | **Goals** | **Activities** |
| --- | --- | --- |
| 1. General information on protein-rich nutrition and resistance exercise | - Participants know that exercise in combination with protein-rich nutrition contributes to maintaining or improving muscle mass and strength; - Participants can name a few examples of protein-rich products. | 1. Welcome 2. Introduction round 3. Introduction protein-rich nutrition 4. Introduction resistance exercise 5. Explain homework assignment and give preview of second workshop |
| 2. Breakfast and introduction ProMuscle mobile application | - Participants can mention protein-rich products that contribute to the protein-content of their breakfast; - Participants can explain how to read nutrition labels and how to define the protein-content of a product based on the label; - Participants are able to make a healthy and protein-rich breakfast; - Participants plan to consume sufficient protein at breakfast at home more often. | 1. Welcome 2. Discuss previous workshop and homework assignment 3. Discuss breakfast 4. Discuss product labels 5. Make breakfast and taste the different dishes 6. Explain homework assignment and give preview of third workshop 7. Explain the ProMuscle mobile application (optional) |
| 3. Lunch | - Participants can mention protein-rich products that contribute to the protein-content of their lunch; - Participants can explain what the labels on products mean; - Participants are able to explain the differences between types of dairy products; - Participants are able to make a tasty and protein-rich lunch; - Participants plan to consume sufficient protein at lunch at home more often. | 1. Welcome 2. Discuss previous workshop and homework assignment 3. Discuss lunch 4. Make lunch and taste the different dishes 5. Explain homework assignment and give preview of fourth workshop |
| 4. Dinner | - Participants can mention protein-rich products that contribute to the protein-content of their dinner; - Participants are familiarised with different types of protein-rich products; - Participants are able to explain the differences between types of dairy products; - Participants are able to make a tasty and protein-rich dinner. | 1. Welcome 2. Discuss previous workshop and homework assignment 3. Discuss dinner 4. Make dinner and taste the different dishes 5. Closure |
| 5. Supermarket visit (optional) | - None specified. | None specified |

**RESISTANCE EXERCISE SESSIONS**

**General key attention points:**

- Create a nice atmosphere during the exercise sessions
- Supervise and motivate participants
- Ensure that participants have faith in the supervision and their own abilities
- Remove fear of performing the exercises
- Build a personal connection with the participants
- Stress that participants exercise for their own health!

**Specific key points for sports hall (no training machines available):**

- Provide progressive resistance-type exercise sessions, so the intensity of exercises should increase (mostly by increasing number of repetitions). Intensity can be also increased with elastic bands or free weights.
- Perform exercises for the major muscle groups, with most emphasis on the leg muscles (20–25 minutes in each training session). Exercises for chest, back, shoulders, and core can be somewhat progressive, but pay attention to individual load ability and participant experiences. The intensity of these exercises should not negatively influence the training intensity of the leg exercises.
  - Leg exercises: 3 sets, 10–15 repetitions, exercises should be moderate to high intensity
  - Other exercises: 15 repetitions, exercises should be of mild intensity.
- Safety in performance of exercises is key. Complex exercises that require balance should be done only under supervision.
- Make sure participants breathe out with concentric movements and breathe in with eccentric movements.
- Ensure sufficient rest between the different exercises.

**Specific key points for fitness centre (training machines available):**

- Provide progressive resistance-type exercise sessions, so the intensity of exercises should increase (mostly by increasing weights or number of repetitions).
- Perform exercises for the major muscle groups, with most emphasis on the leg muscles (20–25 minutes in each training session). Exercises for chest, back, shoulders, and core can be somewhat progressive, but pay attention to individual load ability and participant experiences. The intensity of these exercises should not negatively influence the training intensity of the leg exercises.
  - Leg exercises: 3–4 sets, 10–15 repetitions, exercises should be moderate to high intensity
  - Other exercises: 3 sets, 15 repetitions, exercises should be of mild intensity.
- Ensure that the right number of sets and repetitions is performed.
- Older adults should train at a high intensity for a maximum of 45 minutes per training session.
- Eccentric movement should take 2 seconds, concentric movement 1 second.
- Safety in performance of exercises is key. Complex exercises that require balance should be done only under supervision.

**Structure of a training session:**

1. Warm-up with the group (5–10 minutes). If possible, use game elements to incorporate fun.
   1. Optional for gym: warm-up on an exercise machine if a group warm-up is not possible for participants.
2. Performance of resistance-type exercises. Explain the exercises and let the participants perform them individually, but under supervision.

- For the sports halls: try to include 5 different exercises each week, of which 3 leg exercises (3 sets per exercise, 10 repetitions for the leg exercises, 15 repetitions for the other exercises). Pay attention to balance (use chairs if necessary). Supervise participants if necessary, depending on the participant’s level.
- For the fitness centre: For the leg press and leg extension machine, build intensity from 65% of 1-RM in week 1 to 75% 1-RM in week 12. Upper body exercises should be performed at around 60% of 1-RM.

1. Warm-down with the group; stretching and balance exercises to improve group cohesion.

Supplemental table 1. Overview of behaviour change methods and practical applications used to change the behavioural determinants during the intensive support intervention and the moderate support intervention.

| **Determinant** | **Methods** | **Definition** | **Practical application** |
| --- | --- | --- | --- |
| General methods | Tailoring | Matching the intervention or components to previously measured characteristics of participants | Intensive support intervention:   - Physiotherapist tailors the intensity of the resistance exercises to each participant’s capabilities (based on strength measurement at baseline and in week 6). - Dietitian provides personalised advice to increase protein intake based on current dietary intake and participant’s preferences.   Moderate support intervention:   - Trainers tailor intensity of exercises to the capabilities of the participants. |
|  | Persuasive communication | Guiding individuals and environmental agents towards the adoption of an idea, attitude, or action by using arguments or other means | Intensive support intervention:   - Physiotherapist explains the importance of resistance-type exercises for older adults and being physically active in daily life. Physiotherapist encourages participant to perform the resistance-type exercises. - Dietitian explains the importance of adequate dietary protein intake in combination with resistance-type exercise during the intake and evaluation consultation. Dietitian encourages participant to increase protein intake.   Moderate support intervention:   - Nutrition workshop leader explains the importance of consuming adequate amounts of dietary protein. |
|  | Feedback | Giving information to individuals and environmental agents regarding the extent to which they are accomplishing learning or performance, or the extent to which performance is having an impact | Intensive support intervention:   - Physiotherapist provides positive feedback during the training sessions and during the mid-term evaluation following the midterm strength measurement. Physiotherapist also provides feedback on correct execution of the exercises. - Dietitian provides feedback during evaluation consultation on the extent to which the dietary intake goal is met.   Moderate support intervention:   - Trainer provides positive feedback during training sessions concerning performance of the exercises. - Nutrition workshop leader might provide feedback during the workshops concerning dietary habits or product choices. |
|  | Facilitation | Creating an environment that makes the action easier or reduces barriers to action | Intensive support intervention:   - Resistance exercise sessions and consultations with dietitian are in the municipality where participants live. - Participants receive protein-rich products to incorporate in their diet every week after one of the training sessions.   Moderate support intervention:   - Resistance exercise sessions and nutrition workshops are in the municipality where the participants live. - To ensure continuity, the resistance exercise sessions are preferably scheduled on the same day as the training sessions in the intensive support intervention. - Ideally, the nutrition workshops are at the same location as the consultations with the dietitian during the intensive support intervention. |
|  | Reinforcement | Linking a behaviour to any consequence that increases the behaviour rate, frequency, or probability. | Intensive support intervention:   - Physiotherapist praises the participant for performing the exercises correctly or for increasing intensity.   Moderate support intervention:   - Trainer praises the participant for performing the exercises correctly or for increasing intensity. |
|  | Belief selection | Using messages designed to strengthen positive beliefs, weaken negative beliefs, and introduce new beliefs | - During recruitment, interested older adults are told why resistance exercise and dietary protein intake are important for maintaining or increasing muscle strength. - A short film shown during information meetings depicts other older adults who are performing the resistance-type exercises and who are positive.   Intensive support intervention:   - Physiotherapist convinces participants that they are able to perform the resistance exercises and increase exercise intensity.   Moderate support intervention:   - Nutrition workshop leader shows participants that consuming adequate protein is feasible, by providing examples of protein-rich products and easy protein-rich meal recipes. |
| Perceived behavioural control / Barriers / Action control | Guided practice | Prompting individuals to rehearse and repeat the behaviour various times, discuss the experience, and provide feedback | Intensive support intervention:   - At the start of the intensive support intervention, physiotherapist shows how to do the resistance exercises, explains the exercises, and provides feedback when the participant does the exercises.   Moderate support intervention:  Not applicable. |
|  | Self-monitoring of behaviour | Prompting the person to keep a record of specified behaviours | Intensive support intervention:   - Participants indicate daily on a calendar whether they consumed the protein-rich products.   Moderate support intervention:   - Participants can register on the mobile application (app) how much protein they consumed and whether they performed resistance exercises. |
|  | Goal setting | Prompting planning what the person will do, including a definition of goal-directed behaviours that result in the target behaviour | Intensive support intervention:   - Dietitian explains that the goal is to consume 25 grams of protein at each main meal and discusses with the participant how this can be achieved by changing dietary habits at the main meals.   Moderate support intervention:   - Nutrition workshop leader reminds participants of the goal to consume 25 grams of protein at each main meal and discusses and demonstrates within the group how this can be achieved. |
|  | Set tasks on a gradient of difficulty | Setting easy tasks and increasing difficulty until target behaviour is performed | Intensive support intervention:   - Physiotherapist starts the intensive support intervention with a familiarisation period, using low intensity. Later, the intensity is slowly increased to improve the participant’s strength, based on the training protocol provided.   Moderate support intervention:  Not applicable. |
|  | Planning coping responses | Getting the person to identify potential barriers and ways to overcome these | Intensive support intervention:   - Physiotherapist might discuss barriers to performance of the resistance exercises and how to deal with them. - During the intake and evaluation consultation, dietitian might discuss difficult situations concerning following the dietary advice (e.g. holiday or disliking products) and how to deal with that.   Moderate support intervention:   - Trainers might discuss barriers to performance of the resistance exercises and how to deal with them. - Nutrition workshop leader might discuss barriers to consuming adequate dietary protein and ask participants to think of ways to overcome these barriers. |
| Attitude | Direct experience | Encouraging a process whereby knowledge is created through the interpretation of experience | Intensive support intervention:   - Physiotherapist guides participants in performing resistance-type exercises and, by doing the exercises, participants find that they can perform the exercises and increase training intensity. - Dietitian evaluates protein intake during evaluation consultation and shows participants that they were able to consume sufficient dietary protein.   Moderate support intervention:   - Nutrition workshop leader lets participants practice with reading labels and preparing protein-rich meals. |
|  | Arguments | Using a set of one or more meaningful premises and a conclusion | Intensive support intervention:   - Physiotherapist explains importance of resistance-type exercise for older persons. - Dietitian explains importance of consuming adequate dietary protein in combination with resistance-type exercise.   Moderate support intervention:   - Nutrition workshop leader explains importance of consuming adequate dietary protein. |
| Social support | Stimulate communication to mobilise social support | Prompting communication about behaviour change in order to provide instrumental and emotional social support | Intensive support intervention:   - Physiotherapist encourages interaction between participants by providing a group-based warm-up and/or warm-down and conversation during training sessions.   Moderate support intervention:   - Trainers can facilitate interaction by providing a group-based warm-up or warm-down of the exercise sessions. - Nutrition workshop leader encourages interaction between participants by asking questions and prompting exchange of experiences in the group. |
|  | Provide opportunities for social comparison | Facilitating observation of non-expert others in order to evaluate one’s own opinions and performance abilities | Intensive support intervention:   - Training sessions are in a group, where participants can compare themselves with, or observe, peers performing the same exercises.   Moderate support intervention:   - Training sessions are in a group, participants can still compare themselves with their peers. - Participants share experiences during the nutrition workshops and can either learn from others or be an example to others. |
| Awareness | Framing | Using gain-framed messages, emphasising the advantages of performing the healthy behaviour, or loss-framed messages, emphasising the disadvantages of not performing the healthy behaviour. | - Participants receive information during recruitment stating gain-framed information concerning the benefits of participating in this project.   Intensive support intervention:  Not applicable.  Moderate support intervention:  Not applicable. |
|  | Consciousness raising | Providing information, feedback, or confrontation about the causes, consequences, and alternatives for a problem or a problem behaviour | Intensive support intervention:   - Dietitian discusses the 3-day food diary during the intake consultation, explains the meals at which protein intake can be improved, and discusses solutions with the participant.   Moderate support intervention:   - Nutrition workshop leader provides examples of protein-rich meals and protein-rich products. |
| Knowledge | Discussion | Encouraging consideration of a topic in open informal debate | Intensive support intervention:  Not applicable.  Moderate support intervention:   - Nutrition workshop leader facilitates discussion on nutrition-related topics. |
|  | Elaboration | Stimulating the learner to add meaning to the information that is processed | - Participants receive information on resistance exercises and the nutrition intervention during recruitment, in the information leaflet, or at an information meeting.   Intensive support intervention:   - Physiotherapist might explain about the resistance-type exercises during the training sessions. - Dietitian provides information on nutrition during the intake consultation and the evaluation consultation and, if needed, during the additional contact moment.   Moderate support intervention:   - Nutrition workshop leader provides information on nutrition during the workshops. |
| Habit | Planning coping responses | Getting the person to identify potential barriers and ways to overcome these | See ‘Coping response’ concerning PBC / Barriers / Action Control |
